# Machine learning algorithms can predict emotional valence across ungulate vocalizations

**DOI:** 10.1016/j.isci.2025.111834

**Published:** 2025-01-17

**Authors:** Romain A. Lefèvre, Ciara C.R. Sypherd, Élodie F. Briefer

**Affiliations:** 1 Behavioural Ecology Group, Section for Ecology & Evolution, Department of Biology, University of Copenhagen, 2100 Copenhagen Ø, Denmark; 2 School of Engineering and Applied Sciences, Harvard University, Cambridge, MA, USA

**Keywords:** algorithms, artificial intelligence, bioacoustics, wildlife behavior, zoology

## Abstract

Vocalizations can vary as a function of their context of production and provide an immediate measure of an animal’s affective states. If vocal expression of emotions has been conserved throughout evolution, direct between-species comparisons using the same set of acoustic indicators should be possible. The present study used a machine learning algorithm (eXtreme Gradient Boosting [XGBoost]) to distinguish between contact calls indicating positive (pleasant) and negative (unpleasant) emotional valence, produced in various contexts by seven species of ungulates. With an accuracy of 89.49% (balanced accuracy: 83.90%), we found that the most important predictors of emotional valence were acoustic variables reflecting changes in duration, energy quartiles, fundamental frequency, and amplitude modulation. This approach is critical in the field of emotional communication, where more information is needed to reach a better understanding of the emotional origins of human language. In addition, these results can help toward the development of automated tools for animal well-being monitoring.

## Highlights

• Machine learning achieves 89.49% accuracy in classifying emotions across ungulate calls
• Key features include call duration, pitch, amplitude modulation, and energy quartiles
• Species differences highlight the need for broader datasets across diverse taxa
• Findings suggest universal tools for welfare monitoring, needing further validation

## Graphical abstract

**Figure:**
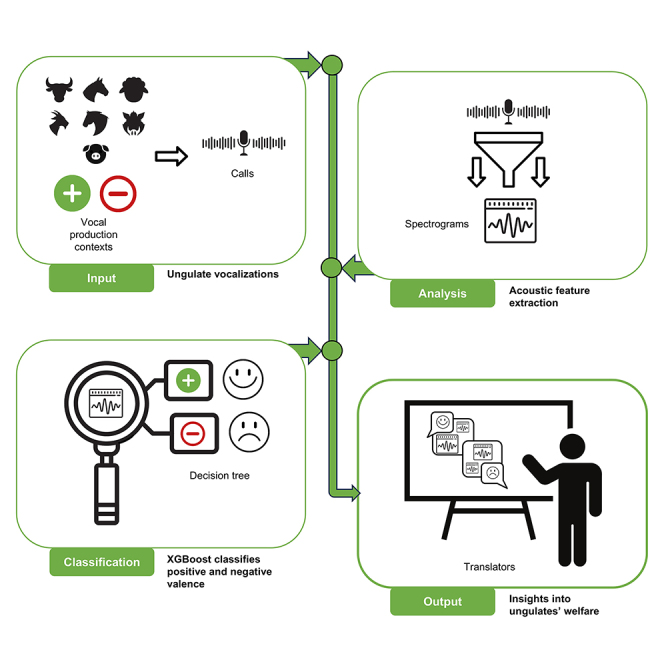

## Introduction

Emotions can be defined as short-lived and vital reactions that elicit changes in the autonomic and somatic nervous systems.^1^ They can be categorized in a two-dimensional space: arousal (bodily activation) and valence (positive/pleasant versus negative/unpleasant).^2^ Emotional arousal is relatively straightforward to infer in non-human animals using, among others, physiological (heart rate or cortisol) or behavioral (body movement) indicators. By contrast, valence is both crucial for animal welfare and notoriously difficult to assess.^3^ However, behavior and, notably, vocalizations are promising valence indicators.^4^

During vocal production, emotion-related changes result in modifications of acoustic features through tension and action of muscles used for voice production.^5^ As a result, consistent changes in vocal expression of arousal have been found across species,^6^ enabling not only humans to recognize emotional arousal in the vocalizations of a wide range of vertebrate species,^7^ but also distant vertebrates such as crocodiles to perceive distress in human baby cries.^8^ It is less clear, however, whether this is also the case for vocal expression of emotional valence.^9^ Yet, few acoustic features (e.g., duration) seem to encode valence across species,^6^ and recent studies suggest that humans can also identify emotional valence above chance levels in the vocalizations of a number of other species.^9^^,^^10^

The recent expansion of computational statistics and their use in detecting and categorizing animal vocalizations widens the potential to decode animal vocalizations.^11^ In recent years, studies that have been interested in sound-emitting species have benefited from the automation of analyses, leading to diverse applications,^12^ such as the detection of vocalizations of endangered bird species for automatic habitat mapping,^13^ conservation status assessment of species,^14^ and automated information retrieval from soundscape recordings.^15^ Recent studies exploring automatic acoustic recognition systems also reported promising results in evaluating the effects of environmental enrichment on separation stress behavior in chicks,^16^ detecting estrus in cattle^17^ and distress in chickens,^18^ or classifying emotional valence in pigs' vocalizations.^19^ However, despite recent advances, machine-driven acoustic classification of emotions has never been explored in multiple species within the same study. The present study represents a first attempt at employing a machine learning algorithm, specifically the eXtreme Gradient Boosting (XGBoost) algorithm, for simultaneous analysis of emotional valence in the vocalizations of multiple species. By analyzing vocal data from several ungulates, we aim to uncover shared acoustic correlates of emotional valence, testing the hypothesis that specific vocal characteristics universally signal positive or negative emotions. This cross-species approach not only distinguishes our work from previous single-species studies but also sets the groundwork for the development of a universal tool for emotional valence classification.

## Results

We performed a series of complementary analyses that aimed at understanding the acoustic correlates of emotional valence in seven species of domestic and wild ungulates: cows, sheep, horses, Przewalski’s horses, pigs, wild boars, and goats^20^ (Table S1 and Data S1). For all these calls, the context of production associated with vocal production was known and allowed us to determine the emotional valence experienced by the animals (validated during previous studies based on behavioral indicators^20^). The limitation to contact calls meant that all calls had the same biological function, hence eliminating this confounding factor. It also ensured a balanced number of low- and high-arousal contexts of each valence, as well as avoided very high arousal contexts such as distress or fear, in order to control for the effect of arousal on vocalizations (Table S1, Data S1). We extracted 17 representative acoustic features from these calls (Data S2 and Table S2), which were used as input variables in our analyses (see STAR Methods for more details).

These analyses aimed to (1) explore and visualize patterns of separability between species and emotional valence categories through dimensionality reduction, using uniform manifold approximation and projection (UMAP), (2) quantify within- and between-species variation in the acoustic features coding for emotional valence, (3) assess the degree of separability of emotional valence for each species using clustering (k-means) and classification (Naive Bayes), (4) achieve automated classification of emotional valence across species using a decision-tree-based ensemble algorithm (XGBoost), (5) identify the most influential acoustic features contributing to valence classification and model predictions through Shapley additive explanations (SHAP), and (6) test the generalizability of the model by relying on cross-validation and species-specific classifiers. The results of these analyses are presented in the following paragraphs, beginning with the exploration of the underlying structure of the acoustic features using UMAP, k-means clustering, and Naive Bayes to assess the separability and classification accuracy of emotional valence within each species, followed by the automated classification of emotional valence using XGBoost, and concluding with the identification of the most important acoustic features driving valence classification through SHAP.

### Context, valence, and species separation with UMAP

First, we relied on a stochastic algorithm (UMAP algorithm) to visually explore and evaluate the degree of separability between the contexts of vocal production, the emotional valence, and the species. UMAP is a dimensionality reduction technique that visualizes complex, high-dimensional data in lower-dimensional space, preserving both local and global data structures.^21^ The primary goal of employing UMAP was to complement our analyses by offering insights into the data distribution, rather than to perform explicit cluster analysis.

**Figure 1:**
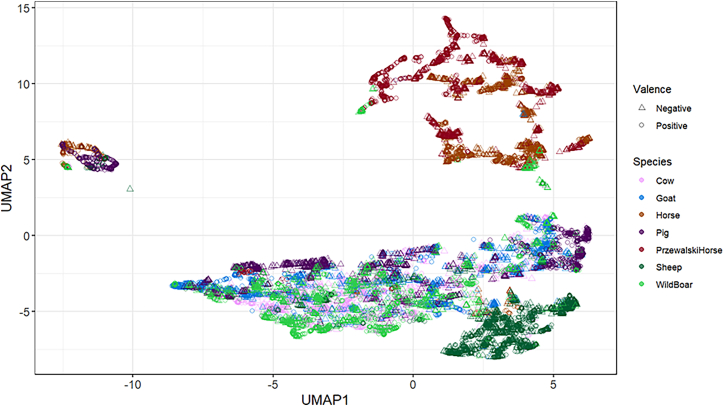
UMAP visualization of emotional valence classification in ungulate vocalizations Valence and species classification based on UMAP mapping. UMAP1 and UMAP2 represent the first and second dimensions, respectively, derived from high-dimensional data to visualize clustering based on similarity. Colors represent the different ungulate species. See also Figure S1 for species-specific visualizations.

Results revealed the separability of positive and negative calls across species (Figure 1). Horses and Przewalski’s horses stood apart from the other species, with their respective clusters being more distant from those of other species. Despite this distance, they both showed clear separability in vocalizations with respect to emotional valence. Sheep vocalizations also stood apart from other species regardless of emotional valence, but showed moderate discrimination between positive and negative valences within their calls. Pig vocalizations formed multiple and scattered clusters with respect to valence, suggesting significant valence separability in their calls. Wild boar calls demonstrated noticeable separation between positive and negative valence, while also showing overlap with other species, indicating both distinct valence separability and potential similarities in emotional expression with other species. Goat calls, on the other hand, exhibited indistinct clusters, with less clear boundaries between positive and negative valence. Finally, cow vocalizations showed moderate overlap between positive and negative valence. Overall, the species’ separation in acoustic features was visible, but the clarity of emotional valence separation varied between species (Figure 1; Figure S1).

Following this visualization, we performed k-means clustering on the UMAP projections for each species to quantify valence separability and calculate clustering purity and classification accuracy using Naive Bayes classifier. K-means clustering was employed as an unsupervised learning method to identify underlying patterns in the UMAP-reduced feature space by grouping vocalizations into clusters that minimize within-cluster variance. This method allowed us to explore natural groupings based on emotional valence within each species, leveraging the reduced dimensionality representation provided by UMAP to enhance interpretability and cluster formation.^22^ In parallel, we applied Naive Bayes classification, a supervised probabilistic algorithm, to the UMAP-projected acoustic features, in order to estimate the probability of vocalizations belonging to positive or negative valence categories. This algorithm is known for its efficiency and effectiveness in datasets where dimensionality is reduced and the features are well defined, such as with UMAP-derived dimensions.^23^ The results of these analyses revealed that pig vocalizations achieved the highest classification accuracy (94.84%) with a confidence interval ranging from 91.68% to 97.60%, and a clustering purity of 69.66% (confidence interval: 63.81%–76.55%). This suggests that pig vocalizations may contain acoustic cues that are reliable enough to allow for successful emotional valence classification and clustering. Similarly, cow vocalizations displayed moderate accuracy (57.18%) but high clustering purity (78.03%), suggesting that, while classification performance is moderate, the high clustering purity still indicates a degree of emotional valence differentiation. In contrast, goat and wild boar vocalizations exhibited lower accuracy rates (49.03% and 49.10%, respectively), with relatively high clustering purity (76.30% for goats and 72.85% for wild boars). The wide confidence intervals for accuracy (goats: 36.54%–61.07%; wild boars: 32.73%–66.42%) suggest that the classifier struggled to predict valence accurately, likely due to overlapping acoustic patterns between positive and negative vocalizations. Similarly, sheep and Przewalski’s horses demonstrated poor classification accuracy (48.25% and 48.03%, respectively), but higher clustering purity (77.65% for sheep and 84.87% for Przewalski’s horses). Specifically, the large confidence intervals for accuracy in Przewalski’s horses (31.93%–62.37%) indicate important variability in the classification model’s performance for this species (Table S3). Overall, these results suggest that while UMAP provides some degree of discrimination, its effectiveness for emotional valence separability may have been influenced by both the complexity of our acoustic data and the intrinsic limitations of the UMAP algorithm in capturing subtle emotional patterns across species.

### Automated valence classification with XGBoost

Following this exploratory phase, we used a decision tree ensemble learning algorithm (XGBoost) to discriminate calls based on their valence. XGBoost is an implementation of gradient-boosted decision trees, and a method used to build a series of trees that are each trained to minimize the residual error and favor the correct classification of cases that were previously misclassified.^24^ Our XGBoost model reported an overall accuracy in classifying valence of 89.49% (confidence interval: 87.31%, 91.41%), a Cohen’s kappa of 0.66, and a balanced accuracy of 83.90% with a sensitivity of 75.00% and a specificity of 92.80%. The model also reported 70.39% of correct classification for the positive calls, 94.21% for the negative calls, and an F-score of 72.62% (Table S4). The confusion matrix reported 126 true positive, 683 true negative, 53 false positive, and 42 false negative classifications.

To strengthen our claims and ensure the identification of universal rather than species-specific features in emotional classification, we additionally trained individual XGBoost classifiers for each species and extracted the ten most important acoustic features based on their gain. This approach enabled us to highlight key features that consistently influenced valence classification across species. Results reported that pigs (99.91%) and Przewalski’s horses (97.78%) achieved the highest model accuracies, suggesting clearer separability between valence categories in these species. Goats, cows, and sheep also demonstrated strong performance, with an accuracy of 90.74%, 92.93%, and 88.55%, respectively. In contrast, species such as wild boars (82.71%) and horses (81.38%) showed lower accuracies, which may suggest more overlapping acoustic patterns, making valence classification more challenging. Based on the ten most important gain values, we found that the interquartile range of amplitude modulation depth (“amEnvDep_iqr”), duration, the 25th percentile of energy quartiles (“quartile25_median”), and the interquartile range of the 75th percentile of energy quartiles for voiced parts (“quartile75Voiced_iqr”) were highly relevant across all species. Additional influential features included the median amplitude modulation depth (“amEnvDep_median”), the median frequency of amplitude modulation for voiced parts (“amEnvFreqVoiced_median”), the interquartile range of amplitude modulation frequency (“amEnvFreq_iqr”), the median fundamental frequency (“pitch_median”), the median frequency modulation (“fmFreq_median”), the interquartile ranges of roughness (“roughness_iqr”) and the spectral centroid (“specCentroid_iqr”), each consistently important across six species. Finally, though less frequent, the interquartile range of fundamental frequency (“pitch_iqr”) and the median roughness for voiced parts (“roughnessVoiced_median”) were influential across five species, while the interquartile range of frequency modulation (“fmFreq_iqr”) appeared in four species (Table S5). While some acoustic features may serve as robust predictors of emotional valence across species, our results suggest that there is also a degree of species-level variability in how emotional states are expressed vocally.

### Model explanation with SHAP

**Figure 2:**
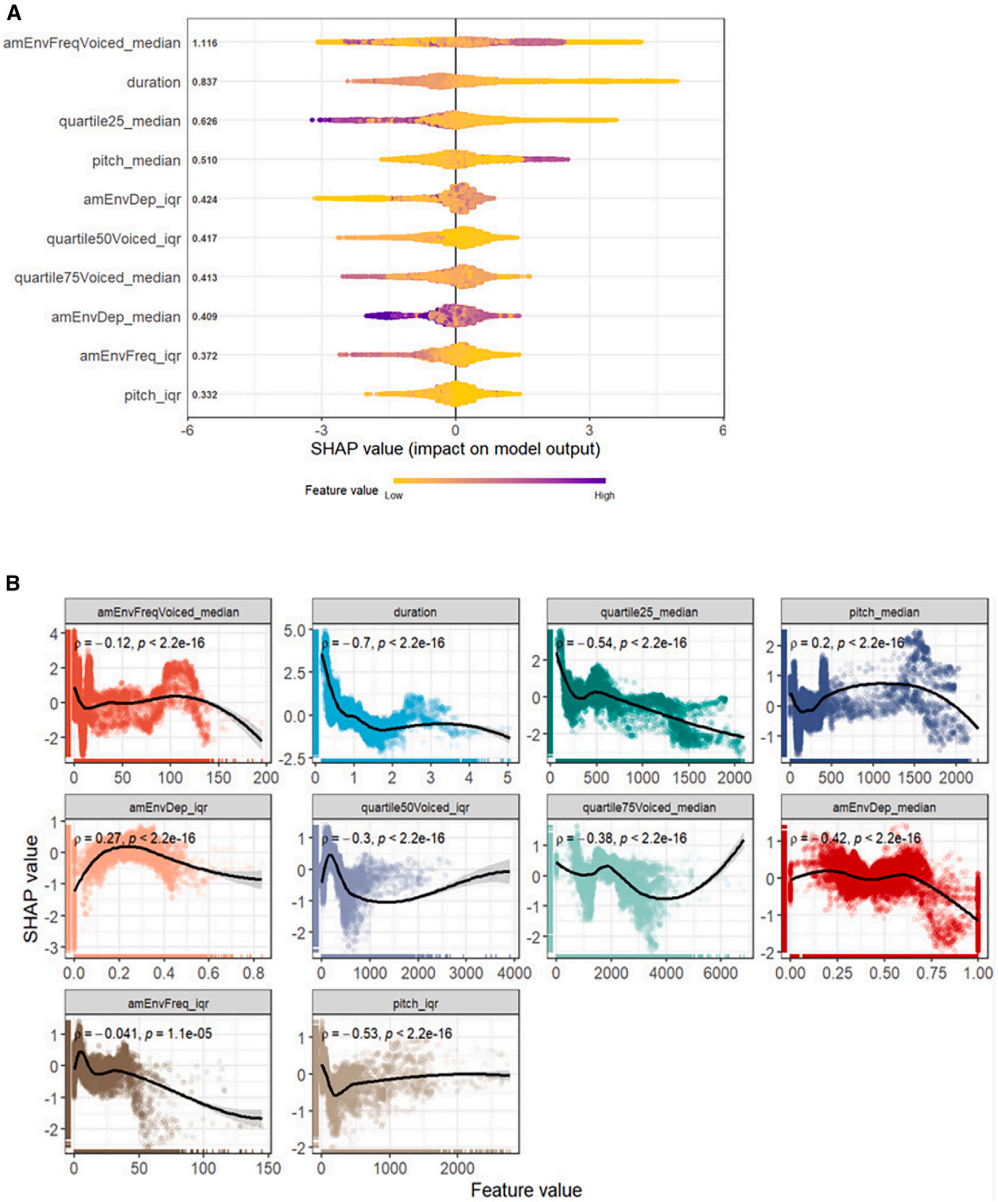
Multivariate analysis of acoustic variables impacting the emotional valence classification in ungulate vocalizations using an XGBoost model (A) Shapley summary plot visualizing the contribution of the ten most important acoustic variables (among 17 extracted variables in total) to the model’s prediction of emotional valence. Each data point on the plot is a Shapley value for a given variable and instance. Acoustic variables are ordered on the y axis according to their mean Shapley value. Shapley values define the direction and magnitude of a variable’s impact on the model’s output. Positive Shapley values indicate that a variable’s value contributes to an increase in the predicted outcome, namely the positive valence, while negative Shapley values indicate a contribution to a decrease in that prediction, pointing toward the negative valence. Color intensity codes for the variable value. (B) SHAP paired correlation plot illustrating the Spearman coefficient correlations (ρ) and *p* value between variable values and their corresponding SHAP values. Positive correlations indicate higher values for a given acoustic variable in positive vocalizations compared with negative ones, while negative correlations indicate higher values for a given acoustic variable in negative vocalizations compared with positive ones.

SHAP values are a method from game theory applied in machine learning to explain model predictions. They quantify the contribution of each variable to a specific prediction, providing detailed insights into model behavior with accuracy and consistency.^25^ We therefore used this approach to evaluate the importance of acoustic variables and their effect on our classifier using SHAP values. Results showed that the ten acoustic variables impacting the prediction of the emotional valence the most in our XGBoost model included variables characterizing the amplitude modulation depth and frequency (“am”), the duration, the energy distribution across quartiles (e.g., “quartile25”) and the fundamental frequency (“pitch”) (Figure 2A).

In complement, we used Spearman’s coefficient correlations to analyze the strength and directionality of each acoustic variable with its respective contribution to the model’s predictions, as indicated by SHAP values. This helped us understand which variables were most influential in classifying emotional valence. Looking at moderate (≥0.30) to strong correlations, positive contact calls exhibited lower depth of amplitude modulation (“amEnvDep_median”), lower fundamental frequency variability (“pitch_iqr”), less spectral energy in high frequencies (“quartile25_median,” “quartile75Voiced_median,” and “quartile50Voiced_iqr”), and shorter duration (“duration”) compared with negative calls (Figure 2B).

## Discussion

The present work provides quantitative evidence for shared acoustic correlates of emotional valence in the contact calls of seven species of ungulates, which could also apply to a broader range of species. Our XGBoost model achieved an overall accuracy of 89.49% in classifying emotional valence, with a balanced accuracy of 83.90%. The model demonstrated high specificity (92.80%) and sensitivity (75.00%), highlighting its effectiveness in distinguishing emotional valence. The detailed insights provided by SHAP revealed the most influential acoustic variables driving these classifications, enhancing our comprehension of the complex interplay between vocal characteristics and emotional expressions. Positive calls were characterized by lower amplitude modulation and lower fundamental frequency variability, contained less spectral energy in high frequencies, and were shorter in duration compared to negative calls. By training individual XGBoost classifiers for each species, we also identified duration and amplitude modulation as acoustic features that consistently ranked highly in their importance to classify valence across species. Overall, our findings hence corroborate the notion that certain acoustic variables may serve as universal indicators of emotional valence across species, supporting previous investigations.^6^^,^^26^ These results support the idea that positive emotional valence is associated with lower energy quartiles, as similarly explored in horses,^27^ Przewalski’s horses,^28^ and wild boars,^29^ as well as less variable fundamental frequency and shorter call duration, two consistent indicators of emotional valence across species.^6^

By training individual XGBoost classifiers for each species, our models demonstrated high overall accuracy in valence classification, with accuracy values ranging from 81.38% in horses to 99.91% in pigs. More importantly, these models confirmed the relevance of key acoustic features such as the interquartile range of amplitude modulation depth (“amEnvDep_iqr”), duration, and energy quartiles (“quartile25_median,” “quartile75Voiced_iqr”), which were identified as important predictors in both individual species classifiers, based on gain, and the general XGBoost model based on SHAP. This alignment between the individual and general XGBoost classifiers strengthens the claim that, while species-specific variability exists, common acoustic correlates may still underpin the vocal expression of emotional valence across ungulates.

UMAP analysis also demonstrated species-specific patterns in acoustic variables and revealed that the clarity of emotional valence separation varied across species. Horses and Przewalski’s horses, grouped together away from other species, both showed significant separability between the two emotional valences. This distinction from other species could be attributed to the fact that horse whinnies can be very long in duration (up to 5 s long) and have a complex frequency modulation.^27^^,^^28^ Sheep vocalizations also set apart from other species and showed moderate discrimination between positive and negative valence. Similarly, pig vocalizations formed multiple and scattered clusters with respect to the two emotional valences, indicating clear separability. This dispersion, with apparent subtypes with respect to emotional valence, could reflect short and long grunts, mostly indicative of positive and negative valence, respectively.^30^ Wild boars’ grunts demonstrated noticeable separation between positive and negative valence as well, but with more overlap compared to pigs’ positive and negative grunts, which showed greater separability, therefore suggesting that the expression of emotional valence could differ between pigs and wild boars, as previously reported.^29^ Goat calls exhibited indistinct clusters with less clear boundaries, indicative of overlapping acoustic variables across different emotional states. Finally, cow vocalizations showed moderate overlap between positive and negative valences.

The application of k-means clustering and Naive Bayes classification to the UMAP projections provided preliminary insights into the separability of emotional valence within each species. Pigs demonstrated the clearest separation, with high classification accuracy (94.84%) and clustering purity (69.66%). This suggests that their contact calls contain distinguishable emotional information, likely due to well-differentiated vocal patterns between positive and negative valence, such as the previously reported distinction between short and long grunts.^30^ In contrast, species like goats, wild boars, and Przewalski’s horses exhibited lower classification accuracy, despite relatively high clustering purity. The variability in performance across species indicates that emotional valence may not be uniformly distinguishable based solely on UMAP projections. While UMAP results indicated that Przewalski’s horses stood apart from other species, the large confidence intervals observed in k-means and Naive Bayes classifications highlight variability in valence-level separability for this species. The high accuracy achieved by the individual XGBoost classifier for Przewalski’s horses may reflect its capacity to capture nuanced acoustic patterns missed by other methods. These differences further highlight the complementary nature of these analyses: UMAP provides a visual overview of patterns of interest, k-means and Naive Bayes offer statistical quantifications, and XGBoost delivers predictive performance by leveraging complex relationships in the data.

Incorporating UMAP for dimensionality reduction provided insightful visualizations of our complex dataset, showing potential relationships in how different ungulate species use similar acoustic features to convey positive and negative affective states. However, the UMAP structures might not necessarily represent distinct biological clusters due to their emphasis on preserving data relationships in reduced dimensions. Interpretations of these visual patterns should be made carefully and be complemented with statistical analyses to ensure that conclusions are grounded in verifiable data due to algorithm limitations. In addition, while the overrepresentation of negative calls in cows could have influenced our results, this potential bias underscores the importance of balanced datasets for training models to ensure generalizability and unbiased performance across various contexts and species.

Our investigation was constrained by the limited availability of validated emotional state datasets, which included seven species. While diverse, this selection highlights the challenges of assembling comprehensive databases across a broader range of species, as well as assessing and validating emotional states under comparable conditions. This limitation highlights the need for increased collaborative efforts to expand these datasets. To this extent, our study serves as a valuable first step, paving the way for future research to further explore the universal aspects of vocal expression of emotional valence across more species. This work should further support the application of machine learning in acoustic communication as a transformative tool for animal welfare research and beyond, with implications for conservation efforts, bioacoustics monitoring, and enhancing human-animal interactions.

### Limitations of the study

While this study provides preliminary insights into the acoustic correlates of emotional valence in ungulates, several limitations should be considered. First, while our dataset encompasses a diverse set of species and achieves high classification accuracy (e.g., 89.49% with XGBoost), the inclusion of additional species and emotional contexts would further enhance the generalizability of our findings. Second, differences in species-specific classification performance (e.g., lower separability in goats and cows) highlight that vocal emotional expression may vary across taxa, and future studies should account for such variability when developing universal models. Finally, the slight overrepresentation of negative calls in some species, such as cows, emphasizes the importance of balanced datasets for training unbiased models. Addressing these limitations in future research will be fundamental to expand the applicability of machine learning tools for understanding emotional communication and improving animal welfare monitoring.

## Resource availability

### Lead contact

For further information and resource requests, please contact Elodie F. Briefer elodie.briefer@bio.ku.dk.

### Materials availability

This research did not produce any new unique materials.

### Data and code availability

• The dataset used in this study (i.e., the sound database) has been uploaded on Zenodo and is publicly available as of the date of publication at https://doi.org/10.5281/zenodo.14636641.
• The original R scripts used for data preprocessing, feature extraction, and model training are not publicly available but will be shared by the lead contact upon request.
• Any additional information required to reanalyze the data reported in this paper is available from the lead contact upon request.

## Acknowledgments

We are grateful to Jeppe H Rasmussen for useful suggestions, Andrey Anikin for his attention and support on using soundgen package as well as for commenting on our manuscript, and Anne-Laure Maigrot, Monica Padilla de la Torre, and Piera Filippi for collecting some of the vocalizations. This research was funded by 10.13039/501100001711Swiss National Science Foundation awarded to E.F.B. (grant nos. PZ00P3_148200 and 310030_185198).

## Author contributions

All authors conceptualized the project. R.A.L. and C.C.S. designed and performed the analyses and wrote the first draft. E.F.B. supervised and funded the project. All authors commented on the manuscript.

## Declaration of interests

The authors declare no competing interests.

## STAR★Methods

### Key resources table

**Table:** 

REAGENT or RESOURCE	SOURCE	IDENTIFIER
**Software and algorithms**		

Praat	Boersma^31^	http://www.praat.org/
R Studio	R Core Team^32^	http://www.rstudio.com/
MATLAB	The MathWorks Inc^33^	http://www.mathworks.com

**Deposited data**		
Zenodo	http://zenodo.org/	Zenodo: https://doi.org/10.5281/zenodo.14636641

### Experimental model and study participant details

This study did not involve human subjects or samples. The data we analyzed were derived from the contact calls of seven ungulate species: cows (*Bos taurus*), goats (*Capra hircus*), horses (*Equus caballus*), Przewalski’s horses (*Equus przewalskii*), pigs (*Sus scrofa domesticus*), wild boars (*Sus scrofa*), and sheep (*Ovis aries*). Details about the species, sex and age, maintenance and care for the animals whose contact calls were used in this study are available in the original studies from which the data were collected^27^^,^^28^^,^^29^^,^^30^^,^^34^^,^^35^^,^^36^ (see also Table S1 and Data S1). All acoustic recordings were collected in accordance with the current laws of the UK (goats) and Switzerland (other species), and approved by ethical committees as part of our previous studies. The experiments carried out to collect the goat recordings were reviewed by the U.K. Government Home Office inspector for Queen Mary, University of London. For the other species, experiments were approved by the Swiss Cantonal authorities (approval numbers: pigs, TG02/2014; wild boars and Przewalski’s horses, ZH011/15; sheep, ZH233/18; horses, VD2689; cattle, ZH49/2014). In total, 3181 contact calls were analyzed across the seven species. The sample size per species and emotional valence is detailed in Table S1 and Data S1.

### Method details

#### Data extraction

We extracted acoustic features of a total of 3181 contact calls collected during our previous studies^27^^,^^28^^,^^29^^,^^30^^,^^34^^,^^35^^,^^36^ (Table S1, Data S1 and S2). None of these collected calls had been produced consecutively by the animals. These vocalizations reflected emotions that were validated as being of either positive or negative valence based on their context of production, such as social interactions versus isolation, behavioral indicators including postural adjustments and movement patterns, as well as physiological measures for domestic species. Each call therefore represented a single instance in the dataset, which was grouped by species, context, and valence during analysis.

Initially, we manually assessed the data quality by listening to the recordings and examining their spectrograms in MATLAB (version R2022b).^33^ During this assessment, 23 calls were identified as having significant environmental acoustic interference, such as overlapping frequencies with other sounds. These calls were excluded from further analysis. The remaining dataset, consisting of 3181 calls, was then manually categorized into two quality levels - 'high quality' and 'moderate quality' - based on the signal-to-noise ratio, both audibly and visually through spectrogram analysis. Then, for the purpose of evaluating the feasibility of including moderate-quality calls in our analysis, we investigated their impact on the performance of a model. This investigation involved 1) training a convolutional neural network, a ResNet-50 architecture, using only the high-quality calls to classify their context of production and species and 2) creating and training a second classifier that included a randomly selected mix of both high- and moderate-quality calls. We then compared the accuracies of these two classifiers to assess if the inclusion of moderate-quality calls had a significant impact on the model’s predictive performance. Our comparative analysis revealed no significant differences in performance between the classifiers trained on high-quality calls alone and those trained on the mixed-quality dataset. This thus suggested that moderate-quality calls maintained sufficient information to contribute meaningfully to our analysis, thereby justifying their inclusion in the combined dataset of 3181 calls for subsequent analysis.

### Quantification and statistical analysis

Acoustic feature processing and statistical analyses were performed in RStudio (version 2022.07.1 + 554).^37^ Amplitude of the calls was normalized prior to extraction with *soundgen* package^38^ and the normalizeFolder() function. The median and interquartile range of 16 acoustic features (i.e., resulting in 32 feature-derived variables) related to frequency, energy, amplitude modulation, noise, and harmonicity were then extracted based on *soundgen* package’s analyze() function. The duration of the calls was also extracted (33 variables in total; Table S6). These acoustic features were chosen among all possible features, as they have previously been shown to vary with emotional valence in the previous studies where these calls were recorded^27^^,^^28^^,^^29^^,^^30^^,^^34^^,^^35^^,^^36^ (Data S1).

For each species, context and valence, acoustic features were extracted based on a 100 ms short-time Fourier transform and a 50 ms sliding window. Only horse and Przewalski’s horse calls were processed using a 50 ms short-time Fourier transform and a 10 ms sliding window to account for rapid variation in the frequency of their calls, as previously reported.^27^ To determine the settings for the automated fundamental frequency (*f*_*o*_) extraction using *soundgen*, we first estimated the *f*_*o*_ range for each species in Praat (version 6.2.14).^31^ To do so, we obtained the minimum, maximum, and mean *f*_*o*_ values using a custom-built script on a balanced dataset consisting of 10 randomly selected calls per species, for each type of context and valence. A similar procedure was used to choose the settings for the frequency range of amplitude modulation, based on the minimum and maximum cumulative variation in amplitude divided by the total call duration (dB/s). Acoustic features were then automatically extracted using *soundgen* with Hanning windowing function^39^ combining autocorrelation, spectral and cepstral methods of estimation of the *f*_*o*_ to determine its contour more precisely. The acoustic data preprocessing involved restricting the frequency range of analysis to between the minimum observed fundamental frequency (*f*_*o*_) of the vocalizations and 20 kHz. This decision aimed to focus on the spectral content most relevant to the animal vocalizations by excluding higher frequencies predominantly associated with background noise, thereby enhancing the signal’s clarity for variable extraction. To determine the fundamental frequency, we performed an automated spectral analysis in *soundgen* to assess the frequency components in each call and identified the lowest frequency at which vocalization energy was consistently present. In addition, we graphically checked the quality of pitch tracking using *soundgen*’s spectrogram() function for 10 randomly selected calls from each species, across each identified context and valence (see examples in Figure S2).

#### Data manipulation

We decided to replace observations affected by missing data (mean ± SD per species = 3.10 ± 2.85%, range = 0–17.50%; Table S7) by 0 using lapply() framework and is.na() function from the *base* package.^32^ This decision aligns with the biological context of our data, as imputation could inadvertently introduce biases that do not reflect the inherent properties of non-voiced vocalizations. Subsequently, to account for species-specific variations in the acoustic feature-derived variables, we normalized them by species based on z-scaling, addressing infinite values and applying natural log-transformation to positive, non-zero numeric variables to reduce skew and enhance comparability using the log() and scale() function from the *base* package.^32^ This ensured that our dataset met the required assumptions for subsequent Uniform Manifold Approximation and Projection (UMAP) and eXtreme Gradient Boosting (XGBoost) analysis. Finally, we addressed potential multicollinearity^40^ based on the Variance Inflation Factor (VIF) method. This approach involved identifying and removing variables with a VIF superior to 5,^41^ and then recalculating the VIFs for all remaining variables by using the vif() function from the *usdm* package.^42^ This iterative process was repeated until all variables had VIFs below our given threshold. This process led to retaining 17 (Table S2) out of the 33 acoustic feature-derived variables initially included after performing recursive VIF.

Then, we performed a stratified train-test split (70/30) for each individual with createDataPartition() function from *caret* package,^43^ ensuring that calls from the same individual were uniquely assigned to either the training or testing sets in order to minimize bias due to individual differences. This approach prevented the subsequent models from learning individual-specific acoustic variables instead of those associated with emotional valence. The final selection of the train-test split and sampling method was further optimized based on the F1-score extracted from the XGBoost classifier across species, as described in subsequent sections, ensuring that the chosen split maximized the model’s performance in distinguishing emotional valence. Then, we oversampled the training set to rebalance the number of calls to be equal between emotional valence and species based on synthetic minority oversampling (SMOTE). SMOTE was chosen for its ability to synthetically balance the class distribution, thereby preserving the integrity of the original dataset, mitigating model bias toward the majority class, reducing the risk of overfitting, and enhancing model generalization. This method ensures a more equitable representation of all classes, which is crucial for the performance of our model in an imbalanced dataset scenario.^44^ Functions used during this process were recipe(), step_smotenc(), prep() and bake() from the *themis*^45^ and *recipes* package.^46^ To ensure consistency, the same training set was used across all analyses, including UMAP, k-means clustering, Naive Bayes classification, and XGBoost models. The test set was used, where relevant, to evaluate model performance. All results reported in this study are also based exclusively on the test set, hence avoiding data leakage from the training process.

##### Context, valence and species separation with UMAP

We relied on the Uniform Manifold Approximation and Projection (UMAP) algorithm to visualize the separability between the species and the valence of call emission. UMAP is a non-linear dimensionality reduction algorithm that seeks to build a uniform representation of the data based on combinatorial structures. It uses Fuzzy K-nearest neighbors distances and spectral embedding to preserve the local and global integrity of the data.^21^ Compared to other widely used dimensionality reduction techniques, such as t-distributed Stochastic Neighbor Embedding (t-SNE), UMAP is a stochastic algorithm that exhibits a stronger emphasis on the global structure of the data to make inter-cluster relationships more meaningful. The distance metric in the UMAP analysis was Euclidean, the number of dimensions was 2, the number of nearest neighbors was 30, and the effective minimum distance between embedded points was 0.001. To perform the UMAP, we used the step_umap() function from *embed* package^47^ to project the variables based on unsupervised learning and ggplot() function from *ggplot2* package^48^ to visualize the species and valence separability (Figure 1) between species, and the valence separability within each species (Figure S1).

Additionally, to quantitatively assess the separability of positive and negative calls, we applied k-means clustering to the UMAP projections for each species using the kmeans() function from the *stats* package.^32^ K-means was used as an unsupervised method to identify clusters based on the acoustic features, without using the true emotional valence labels. To evaluate the quality of the clustering, we computed clustering purity, which measures the proportion of calls in each cluster that belong to the most frequent valence category. Higher purity indicates better alignment between the clusters and the actual valence labels. In parallel, we applied a Naive Bayes classifier, where valence was the response variable and the first and second UMAP dimensions were the predictors, using the naiveBayes() function from the *e1071* package.^49^ Unlike k-means, this is a supervised learning method, which we used to calculate the classification accuracy, by comparing the predicted valence with the actual valence labels. Both k-means clustering and Naive Bayes training set were split using a group-based K-fold cross-validation approach based on the groupKFold() function from the *caret* package,^43^ ensuring that calls from the same individual were assigned exclusively to either the training or test sets. This approach minimized bias from individual differences and prevented models from learning individual-specific acoustic features, focusing on valence-related patterns instead. Additionally, this cross-validation simulated scenarios where models were trained on a subset of individuals and tested on entirely separate individuals within the same species, avoiding data leakage. Finally, we evaluated the statistical robustness of both clustering purity and classification accuracy by calculating 95% confidence intervals using bootstrapping with 1000 iterations, with the quantile() function from the *base* package^32^ to account for variability. During bootstrapping, we resampled the accuracies and purities from the cross-validation folds and calculated the average for each iteration, using the mean values across folds as the final estimate (Table S3).

##### Automated valence classification with XGBoost

We aimed at evaluating the performance of a machine learning algorithm to discriminate the emotional valence (i.e., negative/positive) of our vocalizations by training a XGBoost model. XGBoost takes advantage of parallel processing, tree-pruning, handling missing values, and regularization to provide more accurate approximations that are less prone to overfitting. We chose this algorithm for its efficiency to outperform most other supervised learning algorithms due to its speed and versatility to deal with a wide range of tasks.^50^ To validate the generalizability of our model beyond the specific conditions of our dataset, we also employed a stratified group k-fold cross-validation approach with groupKFold() function from *caret* package.^43^ Each fold contained unique individuals, preventing data leakage between training and testing set. Subsequently, we trained a binary classifier using a 10-fold group cross-validation method to determine the emotional valence of vocalizations from our multiple ungulate species under various contexts. This approach leveraged individual and species-specific identifiers to maintain distinct subsets of data in each fold, thus circumventing the model’s potential bias toward individual- or species-specific characteristics. Hyperparameters were tuned based on grid search optimization technique, in order to test the effectiveness of our model,^51^ using expand.grid() function from the *base* package^32^ to create the grid with potential candidate values, and trainControl() as well as train() functions from *caret* package^43^ to estimate optimal parameters. This step aimed at facilitating a gradual and more robust learning process, reducing the likelihood of overfitting. We set the number of decision trees in the final model to 500, the maximum depth of individual trees to 10 and the learning rate to 0.10 (Table S8). We evaluated model performance based on the trade-off between the true positive rate and the positive predictive value from the Precision-Recall curve (PR-AUC). To provide valid and consistent indicators of emotional valence to be explored in future studies, we used the decision-tree-based ensemble algorithm eXtreme Gradient Boosting (XGBoost) with xgboost() function from *xgboost* package.^52^ Finally, we trained individual XGBoost classifiers for each species separately using the train() function from the *caret* package,^43^ following the same methodology as described for the overall model, including cross-validation and hyperparameter tuning, specific to each species. We then compared the 10 most important acoustic feature-derived variables across species based on their gain, which reflects each feature’s contribution to improving model accuracy, using the xgb.importance() function from the *xgboost* package^52^ (Table S5).

##### Model explanation with SHAP

After modeling the data, we assessed the importance of each acoustic variable and their respective effect on the model by selecting the ten most important contributors based on SHapley Additive exPlanations (SHAP) with *SHAPforxgboost* package^53^ (Figure 2A). SHAP is a method used to explain individual contributions to a particular model output across all possible combinations by considering the local accuracy of variable coalition, variable absence, and consistency to be scored with Shapley values. Model explanation based on Shapley values provides a good alternative to information gain attributes in order to evaluate variable importance in driving the predicted outcomes. In recent years, SHAP has become one of the most efficient tools to make global interpretations more consistent with local explanations and decipher the “black box” of any machine learning algorithm.^25^ In addition, we explored the relationships between acoustic variables and their contributions to model predictions, as quantified by SHAP values. Instead of using straight lines to represent these associations, we used the geom_smooth() function from the *ggplot2* package^48^ to incorporate non-linear trend lines and hence provide a better understanding of the acoustic variables influencing emotional valence classification (Figure 2B).

### Additional resources

No additional resources were created or further expanded as part of this study. Clinical registry numbers and links are not applicable to this research.

## Supplemental information

Document S1. Figures S1 and S2 and Tables S1–S8

Data S1. A spreadsheet with detailed information for each call, including animal ID, species, call type, emotional valence, context of production, number of calls, and references to the studies from which the calls were collected, related to STAR Methods

Data S2. A spreadsheet with a summary of the acoustic features extracted before data processing, including metrics for duration, frequency, amplitude modulation, harmonicity, and other features across species, contexts, and valence categories, related to STAR Methods

Data S3. A spreadsheet with a statistical summary of the acoustic features categorized by species and emotional valence, such as the number of observations, mean, standard deviation, median, minimum, maximum, range, skewness, kurtosis, standard error, and interquartile range, related to STAR Methods

Data S4. Audio recordings of calls from seven ungulate species in positive contexts, related to STAR Methods and Figure S2
