# Supplementary material for: Machine learning algorithms can predict emotional valence across ungulate vocalizations
Source: iScience. 2025 Jan 17;28(2):111834. doi: 10.1016/j.isci.2025.111834 (PMC11847267; doi:10.1016/j.isci.2025.111834)
Supplement: Document S1. Figures S1 and S2 and Tables S1–S8 [file mmc1.pdf]

iScience, Volume *28*

Supplemental information

Machine learning algorithms can predict emotional valence across ungulate vocalizations

Romain A. Lefèvre, Ciara C. R. Sypherd, and Élodie F. Briefer

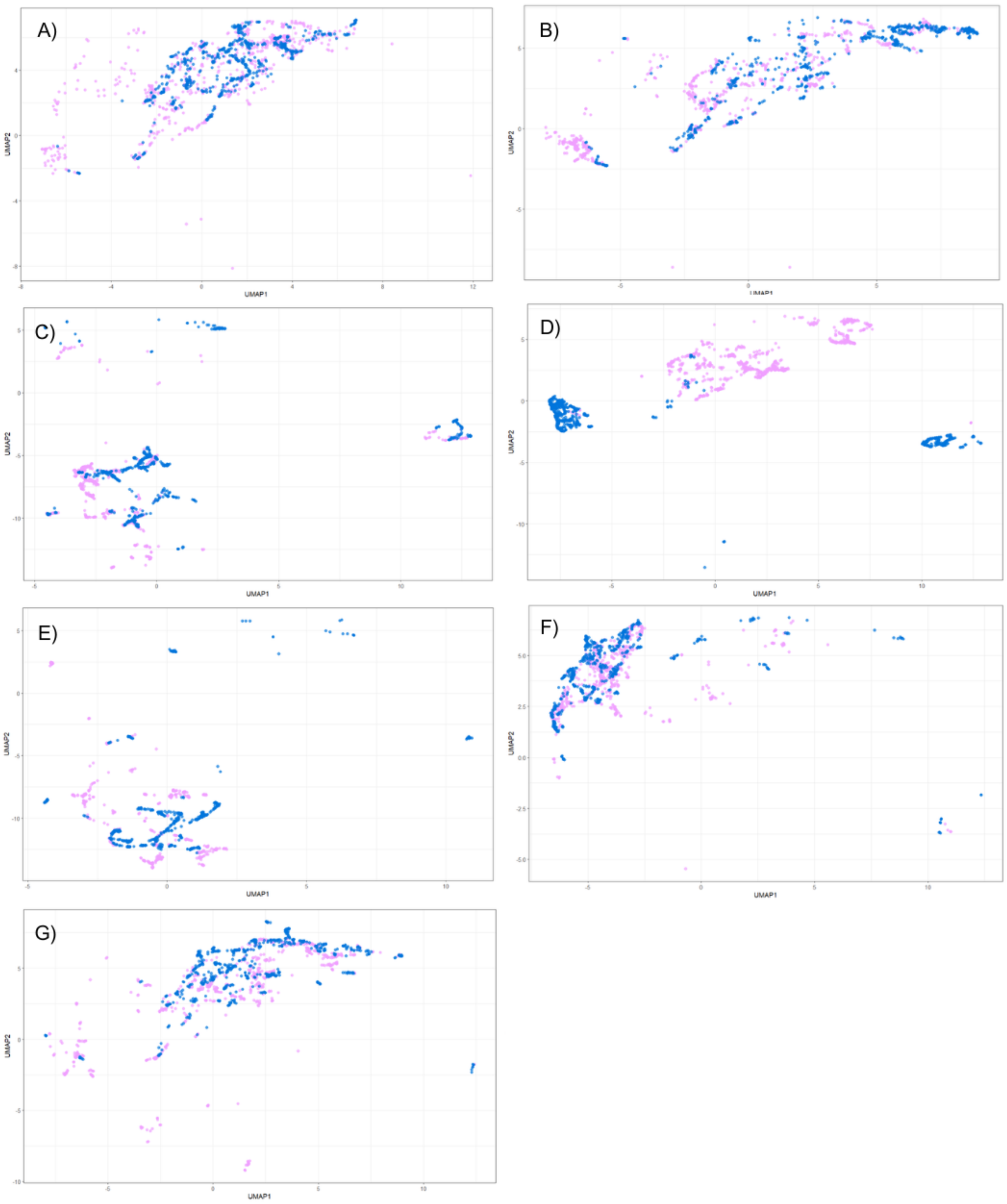

Figure S1. UMAP visualization of emotional valence classification in (A) cow, (B) goat, (C) horse, (D) pig, (E) Przewalski horse, (F) sheep, and (G) wild boar call. Colors indicate emotional valence, with blue representing positive valence and pink representing negative valence. Data are visualized as UMAP projections, which reduce high-dimensional acoustic features to two dimensions for clustering and classification. Related to Figure 1 and STAR Methods.

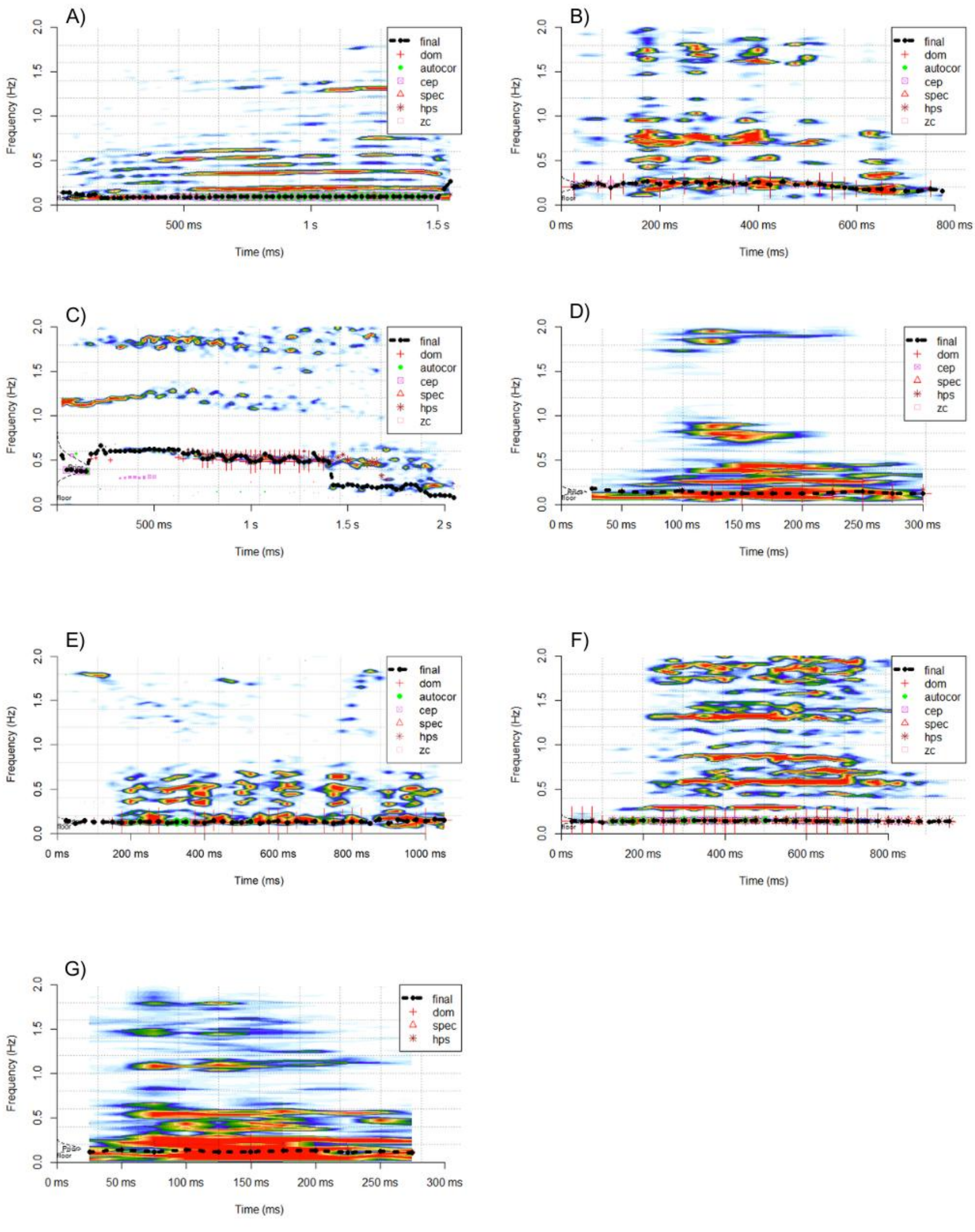

Figure S2. Spectrograms of a (A) cow, (B) goat, (C) horse, (D) pig, (E) Przewalski horse, (F) sheep, and (G) wild boar call. Various pitch estimation methods are overlaid, including the dominant frequency contour (dom), autocorrelation-based (autocor), cepstrum-based (cep), spectrum-based (spec), and harmonic product spectrum (hps), to show the variance in pitch tracking techniques. Zero-crossing points (zc) indicate where the waveform crosses zero amplitude. The overlapping of points suggests convergence in pitch tracking. These sounds are available in Data S4. Related to STAR Methods.

Table S1. Summary of the number of calls according to the species, emotional valence and context of vocal production. Related to STAR Methods.

Species	Valence	Context	Number of calls
Cow	Negative	Full (physical and visual) separation from calf within acoustic range	1078
	Negative	Physical (but not visual) separation from calf	101
	Positive	Physical reunion with calf	21
	Positive	Visual (but not physical) reunion with calf	54
Goat	Negative	Food frustration	80
	Negative	Short social isolation in familiar pen	188
	Positive	Food anticipation	93
Horse	Negative	Separation from one group member	70
	Negative	Separation from the whole group	130
	Positive	Reunion with one group member	16
	Positive	Reunion with the whole group	61
Pig	Negative	Short social isolation in familiar pen	316
	Positive	In pair with food and toys in familiar pen	248
Przewalski's horse	Negative	Group separation for management purpose	30
	Negative	Naturally occurring agonistic interactions	15
	Positive	Food anticipation	19
	Positive	Naturally occurring affiliative interactions	3
Sheep	Negative	Short social isolation in familiar pen	342
	Positive	Reunion with pen mate	54
Wild boar	Negative	Naturally occurring agonistic interactions	158
	Positive	Food anticipation	72
	Positive	Naturally occurring affiliative interactions	32

Table S2. Acoustic feature abbreviations and their descriptions. Each acoustic feature listed in this table has been used as a final predictor of emotional valence in our classifier. Related to STAR Methods.

Acoustic feature	Description
amEnvDep_iqr	Interquartile range of amplitude modulation estimated from a smoothed amplitude envelope, representing the extent of amplitude fluctuations in the sound.
amEnvDep_median	Median depth of amplitude modulation estimated from a smoothed amplitude envelope, representing the extent of amplitude fluctuations in the sound.
amEnvFreq_iqr	Interquartile range of frequency of amplitude modulation estimated from a smoothed amplitude envelope, representing the rate of amplitude fluctuations in the sound.
amEnvFreqVoiced_median	Median frequency of amplitude modulation specifically for voiced parts of the sound, highlighting fluctuations in loudness during vocal cord vibration.
duration	Total duration of the sound in seconds.
fmFreq_iqr	Interquartile range of frequency of frequency modulation (FM), such as vibrato or jitter, in Hz.
fmFreq_median	Median frequency of frequency modulation (FM), such as vibrato or jitter, in Hz.
pitch_iqr	Interquartile range of post-processed pitch contour based on all pitch (fo) estimates, representing the perceived fundamental frequency of the sound.
pitch_median	Median post-processed pitch contour based on all pitch (fo) estimates, representing the perceived fundamental frequency of the sound.
quartile25_median	Median 25th percentile of the spectrum, indicating the frequency below which 25% of the spectral energy is contained.
quartile25Voiced_iqr	Interquartile range of the 25th percentile of the spectrum for voiced frames, focusing on the distribution of spectral energy in voiced sounds.
quartile50Voiced_iqr	Interquartile range of the 50th percentile (median) of the spectrum for voiced frames, focusing on the median frequency of spectral energy in voiced sounds.
quartile75Voiced_iqr	Interquartile range of the 75th percentile of the spectrum for voiced frames, focusing on the distribution of spectral energy in the higher frequencies of voiced sounds.
quartile75Voiced_median	Median 75th percentile of the spectrum for voiced frames, focusing on the distribution of spectral energy in the higher frequencies of voiced sounds.
roughness_iqr	Interquartile range of the amount of spectro-temporal modulation in the “roughness” zone of frequencies, indicative of the perceived harshness or texture of the sound.
roughness_median	Median amount of spectro-temporal modulation in the “roughness” zone of frequencies, indicative of the perceived harshness or texture of the sound.
roughnessVoiced_median	Median amount of spectro-temporal modulation in the “roughness” zone specifically for voiced sounds, focusing on the perceived texture during vocal cord vibration.

Table S3. Clustering purity and classification accuracy for emotional valence separability across species. Data were calculated using k-means clustering on UMAP dimensions and Naive Bayes classification. Purity measures the extent to which clusters align with a single valence category, with higher values indicating better alignment. Accuracy reflects the classifier's ability to correctly identify valence categories. Confidence intervals (CIs) are provided for both metrics, calculated using bootstrap resampling (95% CI). Values above the CI range indicate stronger valence separability and classification accuracy. Data are represented as the mean for each species. Related to Figure 1 and STAR Methods.

Species	Purity	Accuracy	Purity low CI	Purity high CI	Accuracy low CI	Accuracy high CI
Cow	78.03	57.18	67.98	88.27	47.47	67.14
Goat	76.30	49.03	68.36	84.86	36.54	61.07
Horse	74.11	59.92	61.17	85.90	49.73	72.05
Pig	69.66	94.84	63.81	76.55	91.68	97.60
Przewalski’s horse	84.87	48.03	74.31	94.29	31.93	62.37
Sheep	77.65	48.25	65.57	89.57	37.11	61.50
Wild boar	72.85	49.10	63.98	83.40	32.73	66.42

Table S4: Performance statistics for the XGBoost model trained to classify the emotional valence. Accuracy reflects the overall proportion of correct predictions, with a 95% Confidence Interval (CI) calculated using bootstrap resampling. The No Information Rate (NIR) indicates the expected accuracy by predicting the majority class, and the p-value tests whether the model's accuracy exceeds the NIR. Kappa measures agreement between predicted and actual classifications, adjusting for chance. McNemar's Test evaluates significant differences in error rates between classifications. Sensitivity (Recall) quantifies the model's ability to correctly identify positive valence, while Specificity measures correct identification of negative valence. Positive Predictive Value (Precision) indicates the proportion of predicted positives that are true positives, and Negative Predictive Value measures reliability in predicting negatives. The F1 score balances Precision and Recall as their harmonic mean. Prevalence indicates the proportion of positive valence instances in the dataset. Detection Rate represents the proportion of true positives among all observations, while Detection Prevalence reflects the model's tendency to predict positives. Balanced Accuracy averages Sensitivity and Specificity to account for class imbalance. Data are represented as mean ± 95% CI where applicable. Related to STAR Methods and Figure 2A.

Statistics	Value
Accuracy	89.49
95% CI	(87.31, 91.41)
No Information Rate	81.42
P-Value [Acc > NIR]	< 0.0001
Kappa	0.66
Mcnemar's Test P-Value	0.30
Sensitivity	75.00
Specificity	92.80
Positive Pred Value	70.39
Negative Pred Value	94.21
Precision	70.39
Recall	75.00
F1	72.62
Prevalence	18.58
Detection Rate	13.94
Detection Prevalence	19.80
Balanced Accuracy	83.90

Table S5. Variable importance metrics and overall accuracy for the individual XGBoost models trained to classify the emotional valence for each species. Metrics include Gain (the improvement in accuracy attributed to a variable), Cover (the proportion of observations impacted by a variable), and Frequency (the proportion of trees in which a variable is used). Data represent the ten most important acoustic feature-derived variables for each species based on their Gain values. Higher values across these metrics indicate greater influence and frequent utilization of a feature for accurate and reliable classification. Accuracy values are provided for each species, representing the mean ± 95% Confidence Interval (CI) calculated using bootstrap resampling. Species averages for Gain, Cover, and Frequency are also reported to summarize feature contributions across models. Related to STAR Methods.

**Species**	**Acoustic feature-derived variable**	**Gain**	**Cover**	**Frequency**	**Model accuracy**
Cow	amEnvFreqVoiced_median	**0.14**	0.11	0.08	92.93
	amEnvDep_iqr	0.09	0.09	0.08	
	quartile25Voiced_iqr	0.08	0.07	0.06	
	amEnvFreq_iqr	0.07	0.07	0.06	
	specCentroid_iqr	0.06	0.05	0.06	
	duration	0.06	0.08	0.08	
	pitch_median	0.06	0.05	0.05	
	roughness_iqr	0.05	0.06	0.07	
	pitch_iqr	0.05	0.07	0.06	
	quartile75Voiced_median	0.05	0.06	0.05	
**Species average**		**0.07**	**0.07**	**0.06**	
Goat	amEnvFreqVoiced_median	**0.40**	0.18	0.08	90.74
	pitch_median	0.09	0.07	0.06	
	pitch_iqr	0.06	0.07	0.08	
	quartile25_median	0.05	0.06	0.06	
	amEnvDep_median	0.05	0.06	0.06	
	quartile25Voiced_iqr	0.05	0.06	0.06	
	quartile50Voiced_iqr	0.04	0.07	0.08	
	amEnvDep_iqr	0.04	0.06	0.08	
	specCentroid_iqr	0.04	0.05	0.06	
	amEnvFreq_iqr	0.03	0.05	0.06	
**Species average**		**0.08**	**0.07**	**0.07**	
Horse	amEnvFreq_iqr	**0.13**	0.07	0.06	81.38
	amEnvFreqVoiced_median	0.11	0.10	0.06	
	duration	0.09	0.10	0.09	
	quartile25_median	0.08	0.07	0.05	
	roughness_iqr	0.07	0.07	0.07	
	quartile75Voiced_median	0.06	0.05	0.04	
	roughness_median	0.06	0.06	0.07	
	amEnvDep_median	0.05	0.06	0.07	
	fmFreq_median	0.05	0.06	0.05	
	pitch_iqr	0.04	0.04	0.04	

**Species average**		**0.07**	**0.07**	**0.06**	
Pig	quartile25_median	**0.96**	0.85	0.65	99.91
	amEnvDep_iqr	0.04	0.08	0.20	
	quartile75Voiced_iqr	0.00	0.01	0.05	
	pitch_median	0.00	0.01	0.05	
	duration	0.00	0.05	0.05	
**Species average**		**0.20**	**0.20**	**0.20**	
PrzewalskiHorse	amEnvFreqVoiced_median	**0.78**	0.28	0.12	97.78
	quartile25_median	0.06	0.16	0.16	
	roughness_iqr	0.04	0.11	0.12	
	amEnvDep_iqr	0.03	0.07	0.08	
	amEnvFreq_iqr	0.03	0.05	0.03	
	fmFreq_median	0.03	0.04	0.05	
	duration	0.01	0.04	0.05	
	quartile75Voiced_iqr	0.01	0.09	0.14	
	fmFreq_iqr	0.01	0.04	0.05	
	quartile75Voiced_median	0.01	0.03	0.04	
**Species average**		**0.10**	**0.09**	**0.08**	
Sheep	quartile50Voiced_iqr	**0.09**	0.09	0.07	88.55
	amEnvFreqVoiced_median	0.08	0.07	0.06	
	amEnvFreq_iqr	0.07	0.06	0.07	
	duration	0.06	0.06	0.06	
	roughness_iqr	0.06	0.06	0.06	
	quartile25_median	0.06	0.06	0.05	
	amEnvDep_median	0.06	0.07	0.06	
	pitch_median	0.06	0.06	0.07	
	specCentroid_iqr	0.06	0.06	0.07	
	amEnvDep_iqr	0.06	0.07	0.07	
**Species average**		**0.06**	**0.07**	**0.06**	
WildBoar	quartile25_median	**0.12**	0.10	0.08	82.71
	duration	0.11	0.08	0.08	
	pitch_median	0.09	0.09	0.08	
	pitch_iqr	0.07	0.07	0.07	
	amEnvFreq_iqr	0.07	0.07	0.07	
	amEnvFreqVoiced_median	0.07	0.07	0.08	
	amEnvDep_median	0.06	0.07	0.07	
	specCentroid_iqr	0.06	0.05	0.06	
	quartile75Voiced_median	0.05	0.06	0.06	
	roughness_iqr	0.05	0.06	0.06	
**Species average**		**0.07**	**0.07**	**0.07**	

Table S6. Acoustic feature abbreviations and their descriptions, as provided in *soundgen* package documentation[S36]. Every acoustic feature listed in this table with an asterisk (*) has been used as a predictor of the emotional valence in our classifier. For each of them but the duration, we extracted the mean and interquartile range (IQR). Related to STAR Methods.

**Acoustic features**	**Description**
amEnvDep*	Depth of amplitude modulation estimated from a smoothed amplitude envelope, representing the extent of amplitude fluctuations in the sound.
amEnvDepVoiced	Depth of amplitude modulation specifically for voiced parts of the sound, highlighting the extent of loudness fluctuations during vocal cord vibration.
amEnvFreq*	Frequency of amplitude modulation estimated from a smoothed amplitude envelope, representing the rate of amplitude fluctuations in the sound.
amEnvFreqVoiced*	Frequency of amplitude modulation specifically for voiced parts of the sound, highlighting fluctuations in loudness during vocal cord vibration.
duration*	Total duration of the sound in seconds.
fmFreq*	Frequency of frequency modulation (FM), such as vibrato or jitter, in Hz.
Pitch*	Post-processed pitch contour based on all pitch (f_O) estimates, representing the perceived fundamental frequency of the sound.
quartile25*	The 25th percentile of the spectrum, indicating the frequency below which 25% of the spectral energy is contained.
Quartile25Voiced*	The 25th percentile of the spectrum for voiced frames, focusing on the distribution of spectral energy in voiced sounds.
quartile50	The 50th percentile (median) of the spectrum, indicating the frequency below which 50% of the spectral energy is contained.
quartile50Voiced*	The 50th percentile (median) of the spectrum for voiced frames, focusing on the median frequency of spectral energy in voiced sounds.
quartile75	The 75th percentile of the spectrum, indicating the frequency below which 75% of the spectral energy is contained.
quartile75Voiced*	The 75th percentile of the spectrum for voiced frames, focusing on the distribution of spectral energy in the higher frequencies of voiced sounds.
Roughness*	The amount of spectro-temporal modulation in the “roughness” zone of frequencies, indicative of the perceived harshness or texture of the sound.
roughnessVoiced*	The amount of spectro-temporal modulation in the “roughness” zone specifically for voiced sounds, focusing on the perceived texture during vocal cord vibration.
specCentroid*	The center of gravity of the frame’s spectrum, indicating the average frequency weighted by amplitudes. Measures spectral brightness.
specCentroidVoiced	The center of gravity of the frame’s spectrum for voiced segments, focusing on spectral brightness of sounds produced with vocal cord vibration.

Table S7. Number and percentage of missing values per variable derived from acoustic features. Only affected cases have been reported. The Total # of affected cases represents the count of instances where data is missing for a given variable. The Percentage of affected cases indicates the proportion of missing values relative to the total dataset size. Data are represented as counts and percentages for each variable. Related to STAR Methods.

Acoustic feature-derived variables	Total # of affected cases	Percentage of affected cases
fmFreq_median	578	17.50
fmFreq_iqr	578	17.50
amEnvDepVoiced_median	299	8.58
amEnvDepVoiced_iqr	299	8.58
amEnvDep_median	161	4.90
amEnvDep_iqr	161	4.90
specCentroidVoiced_median	142	3.77
specCentroidVoiced_iqr	142	3.77
amEnvFreqVoiced_median	142	3.77
amEnvFreqVoiced_iqr	142	3.77
quartile25Voiced_median	142	3.77
quartile25Voiced_iqr	142	3.77
quartile50Voiced_median	142	3.77
quartile50Voiced_iqr	142	3.77
quartile75Voiced_median	142	3.77
quartile75Voiced_iqr	142	3.77
pitch_median	142	3.77
pitch_iqr	142	3.77
roughnessVoiced_median	142	3.77
roughnessVoiced_iqr	142	3.77

Table S8. Description of optimal parameters we extracted using grid search optimization technique to fit the most accurate XGBoost model. Related to Figure 1 and STAR Methods.

Parameters	Value
nrounds: the number of boosting rounds or trees	500
max_depth: maximum number of nodes	10
eta: learning rate which uses step size shrinkage to prevents overfitting	0.10
gamma: minimum loss reduction required for a node to split further into two more nodes	1.00
colsample_bytree: subsample ratio of variables to use	0.75
min_child_weight: minimum weight required to create a new node	3
Subsample: subsample ratio of the training instances	1.00
